# DECODE: an integrated differential co-expression and differential expression analysis of gene expression data

**DOI:** 10.1186/s12859-015-0582-4

**Published:** 2015-05-31

**Authors:** Thomas WH Lui, Nancy BY Tsui, Lawrence WC Chan, Cesar SC Wong, Parco MF Siu, Benjamin YM Yung

**Affiliations:** Department of Health Technology and Informatics, The Hong Kong Polytechnic University, Hung Hom, Kowloon, Hong Kong

## Abstract

**Background:**

Both differential expression (DE) and differential co-expression (DC) analyses are appreciated as useful tools in understanding gene regulation related to complex diseases. The performance of integrating DE and DC, however, remains unexplored.

**Results:**

In this study, we proposed a novel analytical approach called DECODE (***D***iffer***e***ntial ***Co***-expression and ***D***ifferential ***E***xpression) to integrate DC and DE analyses of gene expression data. DECODE allows one to study the combined features of DC and DE of each transcript between two conditions. By incorporating information of the dependency between DC and DE variables, two optimal thresholds for defining substantial change in expression and co-expression are systematically defined for each gene based on chi-square maximization. By using these thresholds, genes can be categorized into four groups with either high or low DC and DE characteristics. In this study, DECODE was applied to a large breast cancer microarray data set consisted of two thousand tumor samples. By identifying genes with high DE and high DC, we demonstrated that DECODE could improve the detection of some functional gene sets such as those related to immune system, metastasis, lipid and glucose metabolism. Further investigation on the identified genes and the associated functional pathways would provide an additional level of understanding of complex disease mechanism.

**Conclusions:**

By complementing the recent DC and the traditional DE analyses, DECODE is a valuable methodology for investigating biological functions of genes exhibiting disease-associated DE and DC combined characteristics, which may not be easily revealed through DC or DE approach alone.

DECODE is available at the Comprehensive R Archive Network (CRAN): http://cran.r-project.org/web/packages/decode/index.html.

**Electronic supplementary material:**

The online version of this article (doi:10.1186/s12859-015-0582-4) contains supplementary material, which is available to authorized users.

## Background

The identification of complex gene connections and interactions that contribute to the function of living cells is one of the main challenges in functional genomics and system biology. Gene expression profiles provide rich functional information for the study of gene inter-relationships. An early key approach in analyzing gene expression data was based on differential expression (DE). DE analysis has been widely used in many gene expression studies, in which the main task is to identify genes that showed different expression levels across different conditions [1-3]. The motivation is that the differentially expressed genes may have roles in the given phenotypes or conditions, and hence the studying of these genes may reveal the underlying biological mechanisms. In particular, DE analysis is a widely adopted approach that has been successfully applied in cancer research [4-6]. The analysis is useful in prioritising genes that may be dysregulated in cancer. It is popularly used in some challenging problems such as in identifying cancer-specific biomarkers for distinguishing patients and normal subjects, and in identifying potential candidate genes that response to drug treatment and environmental toxins, which will provide illuminative insight on better diagnosis and treatment of diseases at molecular level [4,5,7,8].

However, DE analysis considers each gene individually and their potential interactions are ignored. Biomolecules such as genes, RNAs and proteins do not act alone; they coordinate as functional modules in biological processes and signalling pathways. They also physically aggregate to form nano-machineries such as ribosomes, chaperone and spliceosome to carry out specific functions in the cells [9]. Genes participate in same biological process tend to have similar expression pattern as demonstrated by numerous genome-wide expression studies [10-15]. Furthermore, evidence from previous studies showed that activating a metabolic pathway by small increasing expressions of many genes can be more substantial than a significant over-expression of an individual gene [16,17]. To address the gene independence model in DE analysis, approaches based on gene co-expression, gene sets, and gene clustering have been emerged. They were utilized to explore patterns of RNA expression, and hence intrinsic gene interactions [10-12,18-25].

Extending the gene co-expression concept, the analysis of differential co-expression (DC) has gained much attention in recent years [26-29]. It aims to gain insights into altered regulatory mechanisms between classes, such as disease and healthy controls, by studying their difference in gene co-expression patterns. The analysis is based on the rationale that co-regulated genes tend to share similar expression patterns. As complement to DE analysis, DC analysis is useful in identifying disease genes that may not show significant changes in expressional levels. One possible biological explanation is that given a disease gene, mutations in its coding region or post-translational modifications such as methylation, ubiquitination, and glycosylation, can impair its interactions with other gene counterparts without alternating expression level [26,30].

Evidence from previous studies showed that both DE and DC analyses are useful in identifying functionally important genes. From an informatics perspective, we questioned if relationship exists between these two types of information. Conceptually, if the two approaches extract independent information, we can simply deploy them separately and obtain distinct pieces of information (i.e. two statistically independent gene lists). On the other hand, if they extract dependent information, from a biological perspective, we seek for biological reasons such as cellular functions correspond to such dependency. Furthermore, we evaluated whether combining DE and DC criteria would improve the selection of functional relevant genes. The integrated DE and DC information may provide new opportunities for dissecting complex disease mechanism.

The benefit of integrating DC and DE approaches has been demonstrated by the study of Hudson *et al.* that compared two groups of cattle with or without a known mutation on the transcriptional regulator, namely the *myostatin* [31]. While no significant difference in *myostatin* mRNA levels was found between the two groups, *myostatin* was ranked the most important among 920 transcriptional regulators according to a scoring function that incorporates DC, DE, and expression level. After detailed examination of the scoring system of Hudson *et al.*, we concerned that the differential co-expression term was squared in the score in which the reason was unclear. Moreover, the DE genes were selected using a rather conservative statistical criterion, such that only 85 out of 11,057 genes were identified to be significant.

When integrating DE and DC approaches, one challenging problem is to define appropriate thresholds for selecting high DE genes and high DC gene pairs. Applying over-stringent thresholds may filter out many useful genes and gene pairs; whereas over-relaxing thresholds may lead to high false positivity. This problem is more apparent in DC analysis. Consider an expression data of *m* genes, the number of unique gene pairs is *m(m − 1)/2*. Such huge number of gene pairs makes most multiple testing procedures powerless [32]. As a result, DC gene-pair selection methods were usually based on ad hoc criteria, such as by considering the highest *n%* of gene pairs [27] or by using pre-defined constant thresholds [33].

In this study, we have developed a novel DECODE (***D***iffer***e***ntial ***Co***-expression and ***D***ifferential ***E***xpression) analytical approach that coherently integrates DC and DE aspects. In particular, DECODE aims to improve the identification of functional gene sets or pathways that may be missed out by DC or DE criterion alone. We systematically defined DC and DE thresholds based on the dependency pattern between DE and DC variables. The functional relevance of the identified genes was also evaluated.

## Methods

**Figure 1:**
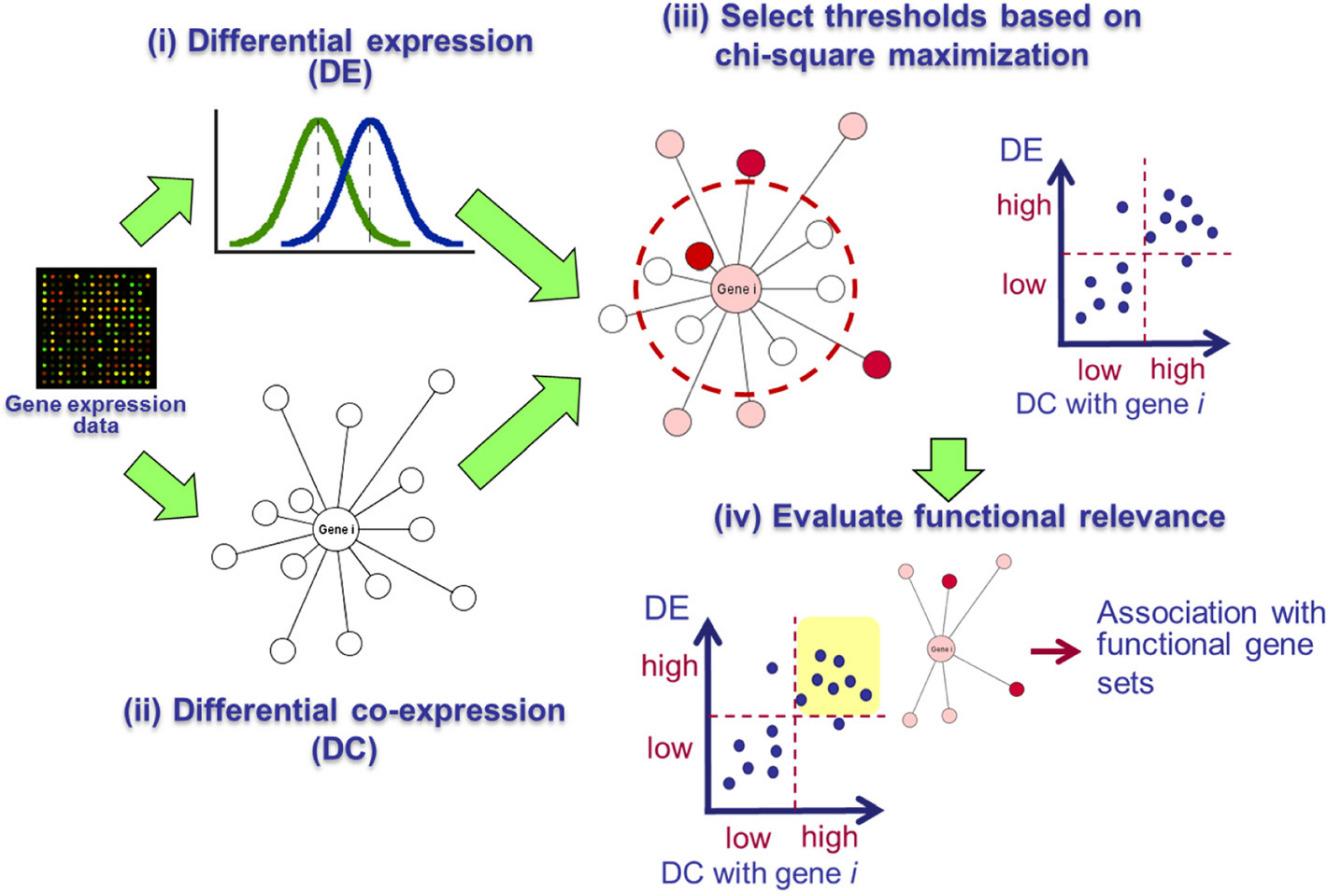
Overview of DECODE. (i) Calculating DE for every gene. (ii) Consider every individual gene i in turn, calculating DC between gene i with every other genes. Genes are represented by nodes. Higher DC between a gene and gene i is illustrated using longer edge. (iii) Selecting optimal thresholds to define high/low DE and high/low DC based on chi-square maximization. Genes with higher DE are illustrated by shading with deeper red colour. (iv) Evaluate functional relevance of selected gene partitions based on functional gene sets.

DECODE consists of four steps: (1) calculating differential expression (DE), (2) calculating differential co-expression (DC), (3) selecting thresholds to define high or low values of DC and DE variables based on chi-square maximization, and statistically evaluating partitions divided by the thresholds, (4) comparing functional relevance of genes categorized into the partitions of high DC, high DE, or both. Figure 1 illustrates the overview of the analytical framework. Details are described in the following sections.

### Differential Expression (DE)

Consider a gene expression data set with *m* genes from samples of two states (or classes): one state consists of case (e.g. disease) group *x*_*D*_, while the other consists of control (e.g. normal or healthy) group *x*_*N*_. We used absolute *t*-value in *t* statistics to quantify the degree of differential expression of each gene. The *t*-value measures the difference of expression levels, in units of standard deviations, between the two states. A positive *t*-value (disease vs. normal) of a gene indicates an up-regulation in disease state; whereas a negative value indicates a down-regulation. A higher absolute *t*-value indicates a larger DE difference. The absolute *t*-value *|t*_*i*_ *|* for a given gene *i*, where *i∈{1,…,m}*, is defined as:

1 $$ \left|{t}_i\right|=\frac{\left|\overline{x_D}-\overline{x_N}\right|}{\sqrt{\frac{{s_D}^2}{n_D}+\frac{{s_N}^2}{n_N}}} $$

where $\overline{x_D}$ and $\overline{x_N}$ are mean expression levels in disease and normal states, *n*_*D*_ and *n*_*N*_ are sample sizes of disease and normal states, and *s*_*D*_ and *s*_*N*_ are standard deviations of expression levels in disease and normal states.

Our current DECODE algorithm has been designed to handle gene expression profiles of large sample size because we have utilized ordinary *t*-statistic to measure DE. In the future, DECODE can be readily modified for expressional analysis of small dataset by incorporating the moderate *t*-statistic [34].

### Differential Co-expression (DC)

We have adopted a widely used differential co-expression measure, *Z* [30,32,35-38]. The *Z* measure quantifies the correlation difference between expression levels of two genes in disease and normal samples. Consider any two genes *i* and *j* in the expression data, let ${r}_{ij}^N$ and ${r}_{ij}^D$ be the Pearson correlation coefficient calculated separately over the samples in normal and disease state, respectively. The measure for differential co-expression, *Z*_*ij*_, between *X*_*i*_ and *X*_*j*_ is defined as:

2 $$ {Z}_{ij}=\frac{\left|{z}_{ij}^N-{z}_{ij}^D\right|}{\sqrt{\frac{1}{n_N-3}+\frac{1}{n_D-3}}} $$

where *n*_*N*_ and *n*_*D*_ are sample sizes in the normal and disease states, ${z}_{ij}^N$ and ${z}_{ij}^D$ are the Fisher-transforms of the correlations for ${r}_{ij}^N$ and ${r}_{ij}^D$, respectively, they are defined as:

3.1 $$ {z}_{ij}^N=\frac{1}{2} ln\left|\frac{1+{r}_{ij}^N}{1-{r}_{ij}^N}\right| $$

3.2 $$ {z}_{ij}^D=\frac{1}{2} ln\left|\frac{1+{r}_{ij}^D}{1-{r}_{ij}^D}\right| $$

After the transformation, ${z}_{ij}^N$ and ${z}_{ij}^D$ are both approximately normally distributed [39,40].

### Novel strategy in selecting optimal DE and DC thresholds based on chi-square (χ^2^) maximization

**Figure 2:**
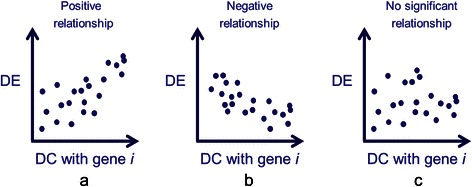
Some possible relationships between differential expression (DE) and the differential co-expression (DC) with gene i. Each point represents a gene. **(a)** Positive relationship **(b)** Negative relationship **(c)** No significant relationship.

With DE and DC measures defined, we investigated the relationship between DE and DC for every gene in the expression data in turn. Given *m* genes in the expression data, there are *m* pairs of relationships between DE and DC for consideration. Specifically, consider an individual gene *i* in the data, we explored the relationship between DE of every gene and DC between gene *i* and every other genes. Figure 2 illustrated some possible relationships using scatterplots.

Next, for each gene *i*, we questioned whether genes with higher DC to gene *i* tends to (or tends not to) have higher DE. To address this, we identify two thresholds for gene *i*. One is used for defining high or low DE; another is used for defining high or low DC. We selected these two thresholds for each gene based on chi-square (*χ* ^*2*^ *)* maximization. In general, a Pearson’s chi-square test is used to evaluate the dependency between two variables. For our purpose, the chi-square test is also used for selecting two optimal thresholds, one for each variable, such that the strongest statistical dependencies between the DE and DC variables can be observed. Defining a variable into three or more categories or comparing chi-square measure with other discretizing measures such as entropy based measure [41] is out of the scope of current study.

**Figure 3:**
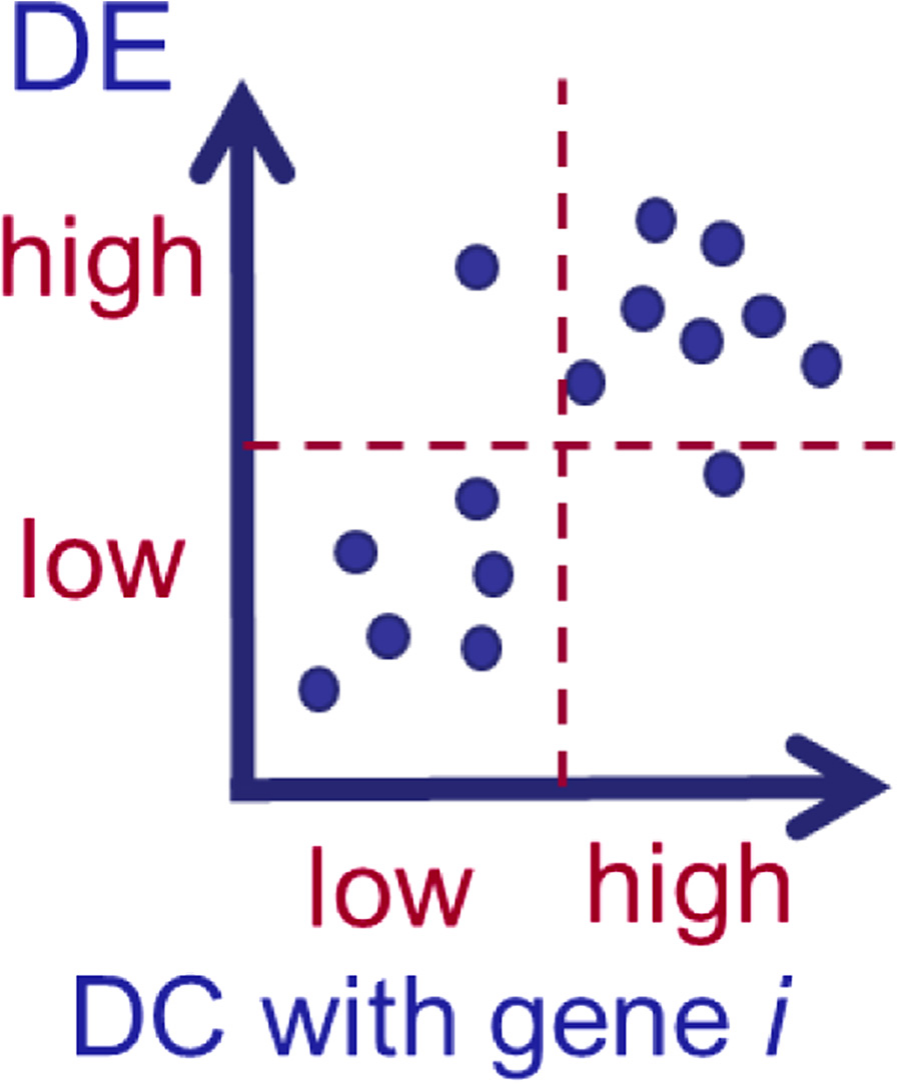
Genes are divided into four partitions based on the optimal DC and DE thresholds. The four divided partitions are (1) low DC and low DE (or LDC_LDE), (2) high DC and low DE (HDC_LDE), (3) low DC and high DE (LDC_HDE), and (4) high DC and high DE (HDC_HDE).

The threshold selection algorithm based on chi-square maximization is described as follows. Given *m* genes in the expression data, for each gene *i*, we seeked for a pair of optimal thresholds, ${z}_i^{*}$ and ${t}_i^{*}$ for the DC and DE variables respectively. The pair of optimal threshold is selected from a set of threshold candidates, *{(z*_*ij*_ *, t*_*j*_ *)}* where *j = {1,…,m}*. Consider each pair of threshold candidates in turn, every gene *k* where *k = {1,…,m}* can be categorized into one of following four partitions as illustrated in Figure 3 including (1) low DC and low DE (or LDC_LDE), denoted as *S*_*LDC_LDE*_, (2) high DC and low DE (HDC_LDE), *S*_*HDC_LDE*_, (3) low DC and high DE (LDC_HDE), *S*_*LDC_HDE*_, (4) high DC and high DE (HDC_HDE), *S*_*HDC_HDE*_. They can be formally defined as:

4.1 $$ {S}_{LDC\_LDE}=\left\{\left({z}_{ik,}\ {t}_k\right),\mathrm{where}\ {z}_{ik}<{z}_{ij}\  and\ {t}_k<{t}_j\right\} $$

4.2 $$ {S}_{LDC\_HDE}=\left\{\left({z}_{ik,}\ {t}_k\right),\ \mathrm{where}\ {z}_{ik}<{z}_{ij}\  and\ {t}_k\ge {t}_j\right\} $$

4.3 $$ {S}_{HDC\_LDE}=\left\{\left({z}_{ik,}\ {t}_k\right),\ \mathrm{where}\ {z}_{ik}\ge {z}_{ij}\  and\ {t}_k<{t}_j\right\} $$

4.4 $$ {S}_{HDC\_HDE}=\left\{\left({z}_{ik,}\ {t}_k\right),\ \mathrm{where}\ {z}_{ik}\ge {z}_{ij}\  and\ {t}_k\ge {t}_j\right\} $$

Based on these four partitions, a two by two contingency table (Table 1) can be constructed in which the number of observed genes in each partition can be counted. The observed frequency for each partition can formally be defined as:

**Table 1:** **2×2 contingency table for DE and DC**

	**Low DC (LDC)**	**High DC (HDC)**	**Marginal total for DE**
**High DE (HDE)**	*obs*_*HDE_LDC*_	*obs*_*HDE_HDC*_	*obs*_*HDE*_
**Low DE (LDE)**	*obs*_*LDE_LDC*_	*obs*_*LDE_HDC*_	*obs*_*LDE*_
**Marginal total for DC**	*obs*_*LDC*_	*obs*_*HDC*_	

5 $$ ob{s}_{A\_B}=\left|{S}_{A\_B}\right| $$

where *A = {low DC, high DC}*, *B = {low DE, high DE}*.

Given the contingency table, the chi-square value, ${\chi}_k^2$, for gene *k* can be computed as follows:

6 $$ {\chi}_k^2={\displaystyle \sum_{A=\left\{ low\ DC,\kern0.75em  high\ DC\right\}}}\kern0.75em {\displaystyle \sum_{B=\left\{ low\ DE,\kern0.75em  high\ DE\right\}}}{\left(\frac{ob{s}_{A\_B}- ex{p}_{A\_B}}{ex{p}_{A\_B}}\right)}^2 $$

where *obs*_*A*_*B*_ and *exp*_*A*_*B*_ are the observed and expected frequency respectively. Assume the two DE and DC variables are independent, the expected frequency can be calculated using the marginal totals of the contingency table (Table 1). They can be computed as follows:

7 $$ ex{p}_{A\_B}=\frac{ob{s}_Aob{s}_B}{m} $$

The pair of threshold candidate, ${z}_i^{*}$ and ${t}_i^{*}$, that gives maximum chi-square value is then selected as the optimal threshold pair for gene *i*. For each gene *i* in the expression data, we perform the same procedure above and obtain their optimal threshold pairs. The chi-square maximization threshold selection procedure can be summarized as follows:

1. For every gene *i*
  (1.1) For every pair of threshold candidates
    (1.1.1) Based on current threshold candidate, all genes can be divided into 4 partitions including
      Low DC and low DE
      Low DC and high DE
      High DC and low DE
      High DC and high DE
    (1.1.2) From the four partitions, construct a 2 × 2 contingency table to count their observed frequencies.
    (1.1.3) Compute the chi-square value based on the contingency table.
  (1.2) Select the threshold candidate pair with maximized chi-square value as the pair of optimal thresholds for gene *i*.

We further evaluated the statistical significance for the association between DC and DE for every gene *i*. For every chi-square value generated in the above procedure, a corresponding *p*-value can also be obtained based on the chi-square distribution. The *p*-values have to be adjusted for multiple testing. First, for every gene *i*, since the chi-square tests are performed for *m* possible threshold candidates, there are *m* tests in total. Here, the *p*-values are adjusted using Bonferroni corrections [42]. Next, since a maximum chi-square value is used for selecting the optimal thresholds for every gene *i*, there are *m* maximum chi-square values in total for comparisons. We further corrected the adjusted *p*-values using a less stringent Benjamini and Hochberg’s method [43]. In later section, we evaluated the false positive control of these adjustments using simulated data.

The chi-square test only examines the significance of the association between DC and DE variables. However, to further evaluate whether the association between high DC and high DE is significant, adjusted residual can be used [44]. If the observed number of genes (formula 5) found in high DC and high DE partition is higher than the expected frequency (formula 7), the association between high DC and high DE is regarded as positive. Conversely, if observed frequency is less than expected, the association is regarded as negative.

**Figure 4:**
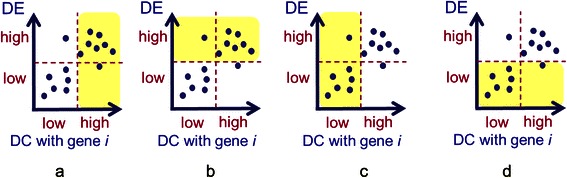
Some possible partitions by combining individual partitions. **(a)** High DC (HDC) only. **(b)** High DE (HDE) only. **(c)** Low DC (LDC) only. **(d)** Low DE (LDE) only.

When the gene partitions are identified based on the optimal thresholds, they provide a flexible framework to study genes with different DC and DE characteristics. For instance, to understand the functional roles of the selected genes in a partition, gene set analysis can be performed. Furthermore, not only these gene partitions can be examined individually, studying on the combinations of partitions is also possible. Figure 4 illustrates some possible combinations. For example, by combining high DC and high DE partition (HDC_HDE) with high DC and low DE partition (HDC_LDE), the resulting partition is high DC (HDC), which can be regarded as a partition selected by using a single or individual high DC criterion.

### Evaluating functional relevance

For a gene partition identified with specific DC and DE characteristics in previous step, we further examined the functional relevance of the partition genes using known functional gene sets. Pre-defined gene sets from Gene Ontology (GO) sets [45,46], Reactome pathways [47] and KEGG pathways [48,49] were used in the current analysis. Two-tailed Fisher’s exact test [50] based on the hyper-geometric distribution is conducted to determine whether a set of partition genes is significantly over-represented in a functional gene set.

To simplify the analysis and interpretation, for each gene partition, only the best associated functional gene set was considered instead of all significant gene sets. Given a partition, the best associated gene set can be defined as the most significant gene set associated to the partition with lowest *p*-value computed from the Fisher’s exact test. The *p*-values have to be adjusted for multiple testing. First, suppose the number of pre-defined functional gene sets is *k*, there are *k* tests between a gene partition and the gene sets. The *p*-values are then adjusted using Bonferroni corrections [42]. Next, consider *m’* selected gene partitions, there are *m’* best associated gene sets. The *p*-values are further adjusted using a less stringent Benjamini and Hochberg’s method [43].

To facilitate the comparison of adjusted *p*-values from different partitions obtained in gene set analysis, a measure, referred to as functional information (*FI*), was used to quantify the significance of association between a gene’s high DC and high DE partition *S*_*HDE_HDC*_ (formula 4.4) and a functional gene set *G*. It is defined as:

8 $$ F{I}_{S_{HDE\_HDC},G}=-lo{g}_2(p) $$

where *p* is the adjusted *p*-value. When the significance of the association is high, *p* is small and in turn *FI* is high.

In this study, we questioned whether the functional information yielded from a partition selected based on the combining criteria of high DC and high DE is higher than that based on either of the individual high DC or high DE criterion alone. For a fair comparison, we considered the thresholds of individual criteria were the same as the optimal threshold pairs, ${z}_i^{*}$ and ${t}_i^{*}$, obtained from the DC and DE criteria for each gene *i* as described in the previous section.

The gain of functional information by combining the high DC and high DE criteria over an individual criterion of DE for a given gene set *G* can be defined as:

9 $$ {\varDelta}_G^{\hbox{'}}=F{I}_{S_{HDC\_HDE},G}-F{I}_{S_{HDE},G} $$

where $F{I}_{S_{HDE},G}$ is the functional information for the association between a high DE gene partition *S*_*HDE*_ and the function gene set *G*.

Similarly, the gain of functional information by combining the DC and DE criteria over an individual criterion of DC for a given gene set *G* can be defined as:

10 $$ {\varDelta}_G^{\hbox{'}\hbox{'}}=F{I}_{S_{HDC\_HDE},G}-F{I}_{S_{HDC},G} $$

where $F{I}_{S_{HDC},G}$ is the functional information for the association between a high DC gene partition *S*_*HDE*_ and the function gene set *G*.

To highlight the functional information gain by combining DC and DE criteria over individual DC or DE criteria for a given gene set *G*, the minimum of individual *FI* gains can be computed using formula 9 and formula 10, which is defined as:

11 $$ {\varDelta}_G^{*}= min\left({\varDelta}_G^{\hbox{'}},{\varDelta}_G^{\hbox{'}\hbox{'}}\right) $$

The minimal *FI* gain is high only when both of the individual gains are high. It is low when any one of the individual gains is low. A negative gain means *FI* based on the combining criteria is lower than either one or both of the individual criteria.

#### Sample size estimation

The method uses three common statistical measures including *t*-statistic, differential co-expression measure based on *z*-transform of correlation coefficient, and chi-square statistics. For *t*-statistics, the sample size requirement depends on factors including alpha-level (*α*), power (1-*β*), and the anticipated effect size (Cohen’s *d*) [51]. For example, consider *α* = 0.05, 1-*β* = 0.8, and *d* = 0.5, the minimum sample size for a two-tailed *t*-test is 128. For differential co-expression measure, consider *α* = 0.05, 1-*β* = 0.8, the difference between two Fisher’s *z* transforms is 0.5, the minimum sample size for a two-tailed *t*-test is 87 [52]. The chi-square test is used to categorized the genes into high/low DC and DE. In applying the test on a 2 × 2 table, the expected frequencies in every cells are required to be greater than 3 or 5. In these examples, the overall minimum sample size required would be 128 given the specification on the expected significant level and power.

## Results and discussion

### Simulation study

The proposed DECODE method provides a way to select thresholds for DC and DE variables for every gene based on chi-square maximization. Based on the maximum chi-square values, the significance of the dependencies between the DC and DE variables were evaluated. The *p*-values were adjusted for multiple testing as described in the method session. We performed simulation to test whether significantly high maximum chi-square values can be generated by chance even when DC and DE were independent. In addition, we evaluated whether the *p*-value adjustment provided good control on false positives rate.

DC and DE variables were simulated for different number of genes (*m*) including 10000, 15000, 20000, and 25000. For each of the *m* genes, we simulated *m* pairs of random *t* and *Z* values for the DC and DE variables respectively. The random *t* and *Z* values are simulated independently. Since DE measure, calculated based on *t*-statistics (formula 1), follows a *t*-distribution, the random *t*-values were generated based on *t*-distribution. The DC measure, calculated based on Fisher-transforms of the correlations (formula 2), are approximately normally distributed [40,53]. Here, the random *Z* values were generated based on normal distribution. All generated *t* and *Z* values were then converted to absolute values. Next, we performed chi-square maximization on these m pairs of DC and DE values.

**Figure 5:**
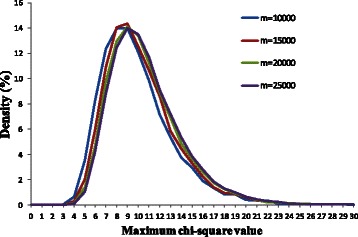
The distributions of the maximum chi-square values for different m number of genes.

The distribution of the maximum chi-square values for different *m* was shown in Figure 5. The average maximum chi-square values and maximum chi-square values at *α = 0.05* were shown in Table 2. Since DE and DC variables were simulated independently, any significant results were regarded as false positives. For example, consider *m* = 10000, the highest 500 chi-square values were false positives at *α = 0.05*. When *p*-values of maximum chi-square values were not adjusted, all 10000 values were significant with confident level of 0.05 as their values greater than the corresponding tabulated value of 3.841.

**Table 2:** **The false positive control using the p-value adjustments on simulated data**

**# of genes (** ***m*** **)**	**Average of maximum chi-square value**	**Observed max. chi-square at α = 0.05**	**# of false positive at α = 0.05**	**# of sig. genes after 1st adjustment**	**# of sig. genes after 2nd adjustment**
10000	11.38	17.70	500	155	4
15000	11.79	17.97	750	215	7
20000	12.10	18.36	1000	231	13
25000	12.27	18.56	1250	280	11

To control these false positives, first adjustment was made for selecting maximum chi-square values from 10000 chi-square values using Bonferroni corrections. This resulted in 155 maximum chi-square values with adjusted *p*-value less than 0.05. In other words, there were 155 false positives. Second adjustment based on Benjamini and Hochberg’s method was then made when comparing 10000 maximum chi-square values. This resulted in only 4 false positives. Results for other values of *m* were shown in Table 2. From the simulation, high maximum chi-square values could be observed because of multiple testing. We showed that the *p*-value adjustments could provide a stringent control on the false positive rate.

### DECODE analysis on breast cancer data

#### Design of experiment

We aimed to systematically determine whether the combining high DC and high DE (or HDC_HDE) criteria outperform individual criteria in selecting functional relevant genes. Specifically, after the best associated gene set was identified for each significant partition and the corresponding functional information was obtained, we evaluated whether the functional information based on the HDC_HDE criteria was higher than that based on individual HDC or HDE criteria.

#### Data sets

Breast cancer data of 25236 genes consisted of 1992 breast tumor samples and 144 normal samples was obtained from European genome-phenome archive [53]. From their study, the tumor data was pre-defined into two random subsets including a discovery set and a validation set. Here, we also randomly split the normal samples into 2 subsets, each with 72 samples. Consequently, we conducted DECODE analysis on two independent sets of tumor and normal samples including a *discovery vs. normal* set and a *validation vs. normal* set. The *validation vs. normal* set was used for evaluating the reproducibility of DECODE in detecting functional gene sets. In addition, to evaluate whether the detection is an artifact, we also performed the same analysis using a *normal vs. normal* set in which both of the case and control groups are the two independent sets of 72 normal samples.

In evaluating the functional relevance of selected genes in the analysis, a total of 7114 functional gene sets were used, including 5895 sets from Gene Ontology (GO) sets (as of Jan 14, 2014) [45], 999 sets from Reactome pathways (release 37) [47] and KEGG pathways (as of July 1, 2011) [48].

#### Overview of the results

In analyzing the *discovery vs. normal* set of the breast cancer data, 17930 genes out of all genes in the breast cancer data have a significant and positive HDC and HDE association (adjusted *p*-value < 0.05). The best associated gene set was then identified for each gene partition of these positive associations. The number of unique best associated gene sets found was 99. For each unique best associated gene set, the mean minimum *FI* gains were calculated.

#### Comparing distribution of average functional information of HDC_HDE partitions in *normal vs. normal* set

**Figure 6:**
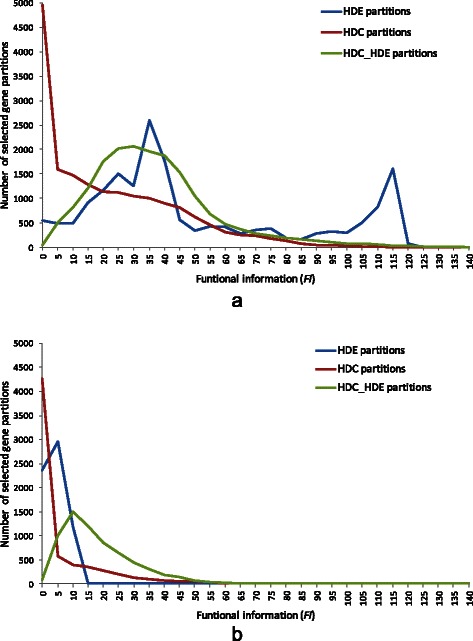
Distribution of function information for different gene partitions using **(a)** discovery vs. normal set, and **(b)** normal vs. normal set.

Among the HDC_HDE partitions of 17930 genes selected from the *discovery vs. normal* set, we investigated the distribution of functional information of their best associated gene sets and compared it to those using individual HDC or HDE criteria. The distributions were shown in Figure 6a. From the figure, a noticeable observation is that when using the HDE criteria, a large group of 1609 partitions were obtained at a high functional information between 120 and 125. Despite of such large group, these partitions were only best associated to two functional gene set including “Cell Cycle (REACT_115566)” and “Cell Cycle, Mitotic (REACT_152)”. In general, the functional information obtained using HDC criteria was lower than HDE or HDC_HDE criteria for these selected partitions.

To determine whether the functional information obtained was an artifact, we performed the same analysis to the *normal vs. normal* set. Out of all genes, only 6870 genes have a significant and positive HDC and HDE association (adjusted *p*-value < 0.05), compared to 17930 in the *discovery vs. normal* set. This difference was expected because the gene co-expressions were less differential when using the *normal vs. normal* set. Figure 6b showed the distribution of the function information of the best associated gene sets obtained for the selected 6870 partitions using different criteria. In comparing Figure 6a and b, the levels of functional information obtained were apparently lower when using the *normal vs. normal* set.

#### HDC_HDE vs. individual HDC or HDE criteria

**Figure 7:**
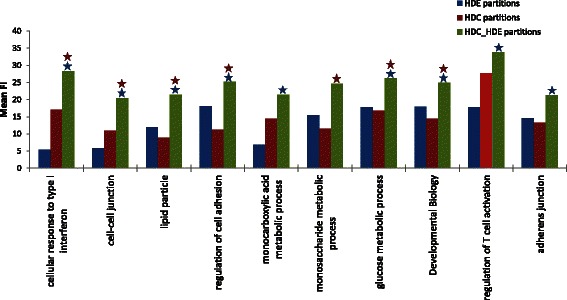
Top 10 best associated gene sets with highest mean minimum functional information (FI) gain, $\overline{\varDelta_G^{*}}$, for HDC_HDE partitions in breast cancer data (discovery vs. normal set). The HDC_HDE partitions (in green) yield significantly higher mean FI than HDC partitions (in red) or HDE partitions (in blue) are marked by red or blue asterisks respectively. The combining HDC_HDE criteria outperformed both of the individual criteria in six gene sets (marked by both red and blue asterisks).

Figure 7 showed the top 10 best associated gene sets with highest mean minimum FI gain for HDC_HDE partitions. More detail results were shown in Additional file 1: Table S1. The combined HDC_HDE criteria outperformed both of the individual criteria in six gene sets, as marked by both red and blue asterisks in Figure 7. An investigation of these gene sets provided useful insights on the mechanisms that are highly altered and highly activated (or inhibited) in breast cancer.

Among the identified gene sets, cellular response to type I interferon (GO:0071357) and regulation of T cell activation (GO:0050863) (Figure 7), are related to the immune response system. Type I interferons are key coordinators of the interactions between tumors and the immune system [54]. They regulate innate and adaptive immune responses such as the activation, migration, differentiation and survival of immune cells including macrophages, monocytes, NK cells, dendritic cells, B cells and T cells [55]. Furthermore, type I interferon response also plays an important role in preventing breast cancer spread to the bone [56].

Cell-cell junction (GO:0005911), regulation of cell adhesion (GO:0030155), and adherens junction (GO:0005912) (Figure 7) are closely related to metastasis in cancer. Metastasis is the process by which cancer spreads from the place of a primary tumor to distant locations in the body. The cell adhesion molecules play a crucial role in metastasis by promoting cell-cell interactions between tumor cells and the endothelium in distant tissues [57].

Lipid particle (GO:0005811), monocarboxylic acid metabolic process (GO:0032787), monosaccharide metabolic process (GO:0005996), and glucose metabolic process (GO:0006006) are related to lipid and glucose metabolism in breast cancer. In breast cancer, metabolisms including lipid and glucose metabolic processes are rewired [58,59], which happen as a result of mutations in cancer genes and alterations in cellular signalling [59]. A well-known metabolic rewiring in cancer is an increase of glucose uptake but a decrease in the proportion of glucose oxidized [60]. These rewired cancer metabolisms maintain the fitness of tumour cells for rapid proliferation and growth [59].

An increased understanding of these innate immune triggers, metastasis mechanisms, and cancer metabolisms can be important in developing new therapeutic strategies aimed at promoting immune responses against tumors, preventing metastasis, and targeting metabolisms in cancer cells. Remarkably, our proposed method was useful in detecting these functions that exhibit high DC and high DE characteristics in breast cancer. The detection on these functional gene sets based on combining criteria outperformed that based on individual high DC or high DE criteria alone.

#### Detecting association between *Type I interferon* and *TRIM22*

**Figure 8:**
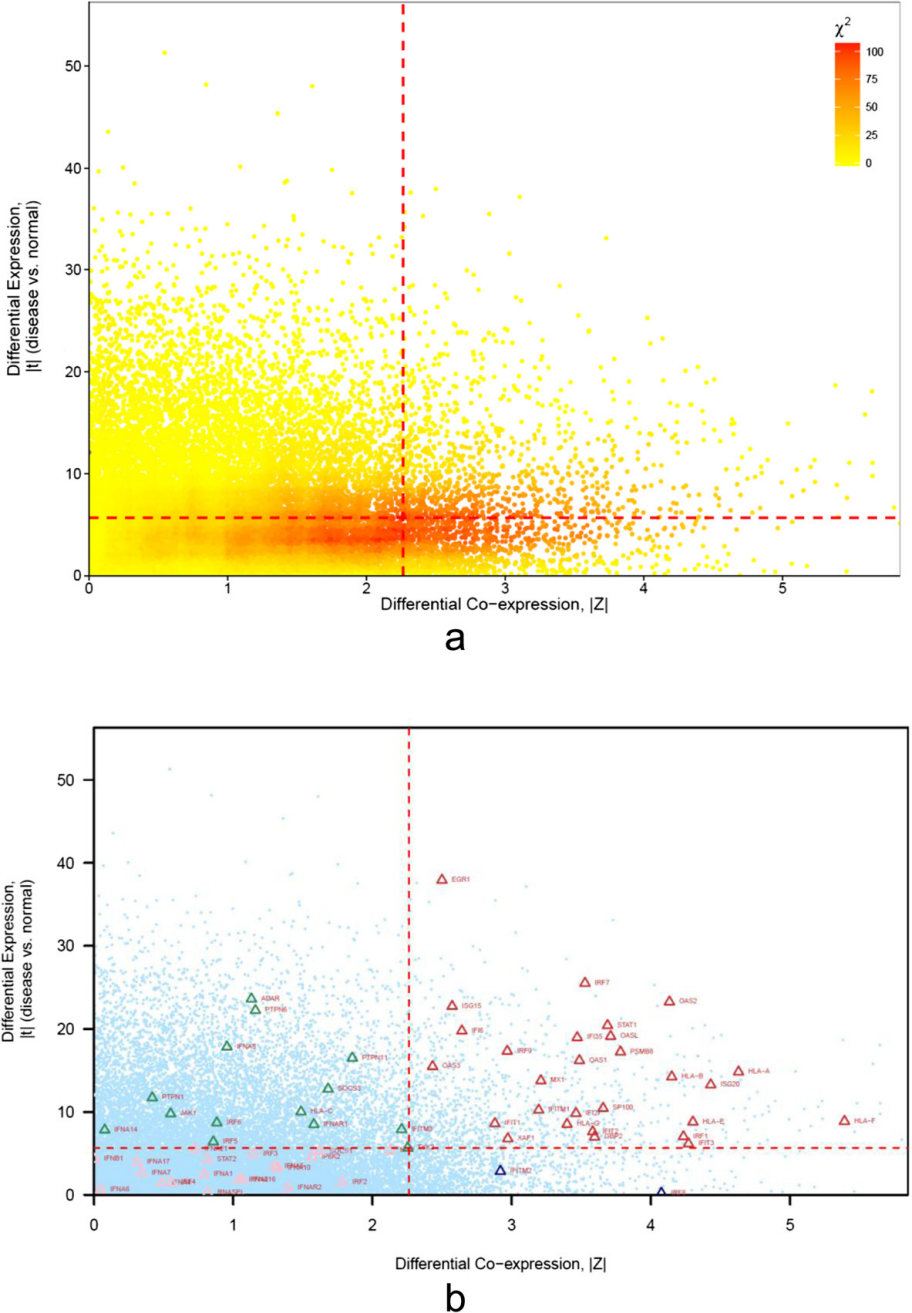
The scatterplot of DE and DC for TRIM22. Each point in the plot represents a gene. The x-axis represents the absolute value of DC, |Z|, between a gene and TRIM22. The y-axis represents the absolute value of DE, |t|, of a gene. The optimal thresholds for DC and DE are drawn using red dash lines. **(a)** Heat map of the chi-square values (χ^2^) for the threshold candidates. **(b)** Gene set “cellular response to type I interferon (GO:0071357)” is best associated to the HDC_HDE partition (Adjusted p-value =2.73 × 10^−18^). Genes found in “cellular response to type I interferon” are highlighted using triangles. These genes with different DC and DE values are highlighted in different colours: high DC and high DE (red); high DC and low DE (blue); low DC and high DE (green); low DC and low DE (pink).

**Figure 9:**
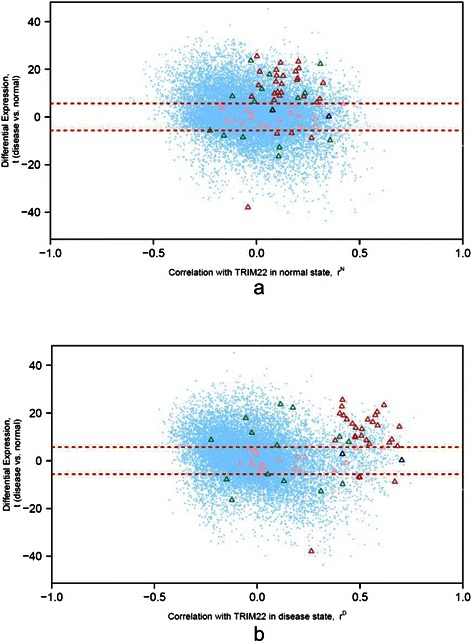
The scatterplot of differential expression (DE) and correlation between TRIM22 and every gene in **(a)** breast cancer state and **(b)** in normal state. Each point in the plot represents a gene. The x-axis in **(a)** and **(b)** represents the correlation coefficients, r^N^ and r^D^, between TRIM22 and every gene in breast cancer state and normal state respectively. The y-axis represents the DE, t, of the gene. A positive t value indicates a higher gene expression in disease state in compare to normal state, and vice versa, a negative t value indicates a lower gene expression in disease state in compare to normal state. The optimal threshold for DE is drawn using red dash line. Genes found in “cellular response to type I interferon” are highlighted using triangles. Similar to Figure 8, these genes with different DC and DE values are highlighted in different colours: HDC_HDE (red); HDC_LDE (blue); LDC_HDE (green); LDC_LDE (pink).

**Figure 10:**
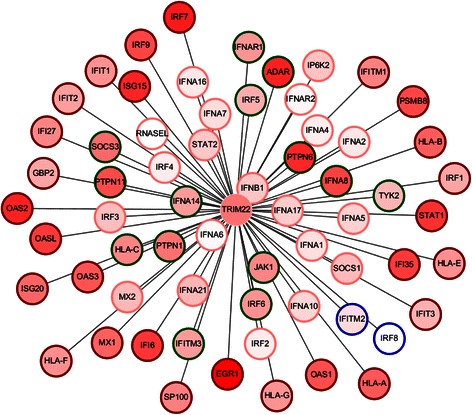
The network betweenTRIM22 and genes of cellular response to type I interferon. Higher DC between a gene and TRIM22 is reflected using longer edge. Genes with higher DE are shaded using deeper red colour. Genes with different DC and DE values are circled with different colours: HDC_HDE (red); HDC_LDE (blue); LDC_HDE (green); LDC_LDE (pink).

Next, to illustrate the DC and DE analysis in more detail, we selected the first ranked best association gene set for further exploration. As shown in Figure 7, the first ranked gene set was “cellular response to type I interferon”. It was the best associated gene set of a total of 27 HDC_HDE partitions. Among these partitions, those of the gene *TRIM22* attained highest minimum *FI* gain of 20.5. Specifically, the gene set was associated to the HDC_HDE, HDC, and HDE partitions with the adjusted *p*-values of 2.73 × 10^−18^ (*FI* = 58.3 bits), 4.18 × 10^−12^ (*FI* = 37.8), and 1.85 × 10^−2^ (*FI* = 5.8) respectively. The average expression of *TRIM22* in disease state was significantly lower than that in normal state with FDR of 4.54 × 10^−22^. It ranked 3166 among 18007 significant differential expressed genes (FDR <0.05). Figure 8 showed the scatterplots of DE and DC for *TRIM22*. The optimal thresholds were selected based on chi-square maximization. The optimal thresholds for DC and DE were 2.263 and 5.654 respectively, which were represented using red dash lines in Figure 8. Figure 8a showed a heatmap for the chi-square values for each pair of threshold candidates. The optimal point was placed in the region of the high chi-square values. The high chi-square values were more spread horizontally along the DC than vertically along the DE dimension. It may implicate a narrower range of DE for detecting high DC and DE dependency in this case. With the optimal thresholds, genes were divided into four partitions including HDC_HDE (999 genes), HDC_LDE (1090 genes), LDC_HDE (8403 genes), and LDC_LDE (14744 genes). The number of genes in HDC_HDE (999) was significantly more than the expected number (778.3) with adjusted *p*-value of 7.49 × 10^−21^. Genes of “cellular response to type I interferon” in these four partitions were highlighted using triangle as shown in Figure 8b. The scatterplot of differential expression (DE) and correlation between genes and *TRIM22* in breast cancer state and in normal state were shown in Figure 9a and b respectively. Most selected genes in HDC_HDE partition, colour in red, were more positively correlated with *TRIM22* in the breast cancer state (Figure 9b) in compare to the normal state (Figure 9a). Twenty-three out of twenty-seven (85.2%) selected genes in the HDC_HDE partition attained a higher expression in disease state whereas the remaining four genes attain a higher expression in normal state. A network between *TRIM22* and the genes of cellular response to type I interferon was shown in Figure 10.

*TRIM22*, tripartite motif-containing 22, previously known as Staf50 (stimulated transacting factor 50 kDa), is a member of the tripartite motif (TRIM) subfamily of RING finger proteins. *TRIM22* underwent self-ubiquitination *in vitro* and *in vivo*, suggesting its functional role as a RING finger E3 ligase [61]. Remarkably, the identified relationship between *TRIM22* and type I interferon was coherent to previous experimental finding [62-65]. *TRIM22* was reported to be inducible by type 1 IFN *in vitro* [62,65]. The association between *TRIM22* and type 1 IFN expression *in vivo* was recently identified in HIV studies [63,64]. *TRIM22* was suggested as an antiviral effector *in vitro* and *in vivo* [63,64]. The expression of *TRIM22* was found to be negatively correlated with plasma HIV viral load but positively correlated with CD4-cell counts in primary HIV-1 infection. Silencing of *TRIM22*, in the presence of IFN-α, could increase HIV infection and virus release. These evidences supported the immune pressure of *TRIM22* against HIV-1. Moreover, *TRIM22* is a p53 target gene and contribute to viral defence by restriction of viral replication [66]. Although the promoter region of *TRIM22* is not p53-responsive, a p53-responsive motif is located in intron 1 of *TRIM22*. The over-expression of *TRIM22* can moderate the clonogenic growth of leukemic U-937 cells suggests an antiproliferative role of leukemic cells. Since *TRIM22* is inducible by both p53 and type I IFN, it may involve in the crosstalk of p53 related pathways and interferon pathways. In short, we demonstrated that the proposed method can generate hypothesis on the relationship between a gene and its associated functional gene sets with high DC and high DE characteristics, plausibly implicated some rewired biological functions in breast cancer for follow-up investigations.

### Evaluation the stability of DECODE using validation set

To evaluate the reproducibility of DECODE, we analyzed the *validation vs. normal* set of the breast cancer data. Out of all genes, 19302 genes were found to have a significant DC and DE association (adjusted *p*-value < 0.05). The best associated gene set was then identified for each gene partition of these positive associations. The number of unique best associated gene sets was 88. Additional file 1: Figure S1 showed the top ten best associated gene sets with highest mean minimum FI gain in using high DC and high DE criteria. The detail of the results was shown in Additional file 1: Table S2 in which the top 30 results were included.

**Figure 11:**
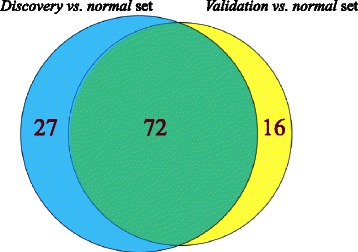
Venn diagrams in comparing the number of selected functional gene sets from the discovery vs. normal set and validation vs. normal set of the breast cancer data using the high DC and high DE criteria. The numbers of common functional gene sets, those found in the discovery vs. normal set, and those found in the validation vs. normal set are shown within the green, blue, yellow shaded areas respectively.

Figure 11 showed the venn diagram in comparing the number of selected gene sets from the discovery set and validation set for the high DC and high DE criteria. Considering the HDC_HDE partitions, 72 unique best associated gene sets were commonly identified, which corresponded to 72.7% of the 99 gene sets and 81.8% of the 88 gene sets identified from the *discovery vs. normal* and *validation vs. normal* data sets respectively. Overall, it showed that a substantial number of functional gene sets identified by the method were reproducible from using two independent data sets of large samples.

### DECODE analysis on Malaysian breast cancer data

In additional to the European breast cancer data obtained from European genome-phenome archive, we have performed the same analysis on an independent set of breast cancer data. The Malaysian cancer data of 13210 genes consisted of 43 tumor samples and 43 normal samples was obtained from GEO Database, accession number GSE15852 [67]. By comparing the results obtained from the European and Malaysian data, common re-wired mechanisms in breast cancer among patients from these two nationality groups may be revealed.

By using DECODE, 8780 genes out of all genes in the data have a significant and positive HDC and HDE association (adjusted p-value < 0.05). The best associated gene set was then identified for each gene partition of these positive associations. The number of unique best associated gene sets found was 54. For each unique best associated gene set, the mean minimum FI gains were calculated.

Additional file 1: Figure S2 showed the top 10 best associated gene sets with highest mean minimum FI gain for HDC_HDE partitions. Detail of the results were shown in Additional file 1: Table S3. The combining HDC_HDE criteria outperformed both of the individual criteria in ten gene sets, as marked by both red and blue asterisks in Additional file 1: Figure S2. Among the top 10 identified gene sets, cellular lipid catabolic process (GO:0044242), monosaccharide metabolic process (GO:0005996), and glycerolipid catabolic process (GO:0046503) are related to the lipid and glucose metabolism, which is consistent to the results obtained from the European breast cancer data. The finding hence indicate the importance of the lipid and glucose metabolic processes in relation to breast cancer.

## Conclusions

We presented a novel method named DECODE as a mean to integrate the DC and DE analysis. DECODE provides an analytic framework for studying different DC and DE characteristics of the genes. By incorporating dependency between DC and DE, high or low values of the DC and DE variables are systematically defined by selecting optimal thresholds that maximize the chi-square value. In using the optimal thresholds, genes can be divided into partitions with different DC and DE characteristics. The statistical significance of a gene partition can further be evaluated by residual test. Noteworthy, since the identified gene partitions at this stage are not constrained or depended on any predefined functional modules or pathways, they provide the opportunities for the discovery of novel disease related genes.

DECODE is useful for investigating whether the functional information of an identified gene partition using the combining DC and DE criteria is higher than that using individual DC or DE criteria alone. In other words, it may generate critical novel biological insights which may not be easily obtained using individual DC or DE approach. In applying DECODE to the breast cancer data, we demonstrated that it can improve the detection of some immune system, metastasis, lipid and glucose metabolism related gene sets using high DE and high DC criteria. Further investigation on the identified gene partitions and the associated functional pathways provides a more systematic understanding of complex disease mechanism, which in turn yields useful insights in the development of new therapeutic strategies for the disease. In conclusion, in complementing the DC and DE analysis, DECODE is a valuable methodology in identifying functional gene sets exhibiting certain combination of DE and DC characteristics, which serves as a new tool for future gene expression studies.

## Additional file

Additional file 1: **Supplementary Materials.**
