# Supplementary material for: DECODE: an integrated differential co-expression and differential expression analysis of gene expression data
Source: BMC Bioinformatics. 2015 May 31;16:182. doi: 10.1186/s12859-015-0582-4 (PMC4449974; doi:10.1186/s12859-015-0582-4)
Supplement: Supplementary file 1 — Supplementary Materials. [file 12859_2015_582_MOESM1_ESM.pdf]

Supplementary Text S1: *Rationale in choosing Bonferroni, Benjamini and Hochberg's methods for the two steps of multiple test adjustment in DECODE.*

Both Bonferroni (more stringent) and Benjamini and Hochberg's (or abbreviated as B&H) (less stringent) methods are commonly used corrections for multiple testing. In our method, two steps of multiple test adjustment were required. First, for every gene i, since the chi-square tests were performed for m possible threshold candidates, there were m tests in total. The first adjustment was made here. Next, since a maximum chi-squared value was used for selecting the optimal thresholds for every gene i, there were m maximum chi-squared values in total for comparisons. The second adjustment was made here.

We tested all four possible combinations of multiple testing adjustment methods using simulated data of 25,000 genes (m=25000) as described in (Manuscript, pg. 14). For each combination, we counted the number of significant genes, which represented the false positives. The result is summarized as follows.

	1st adjustment	2nd adjustment	Number of significant genes (or false positives)
Combination 1	B&H	B&H	24992
Combination 2	B&H	Bonferoni	11
Combination 3	Bonferoni	B&H	11
Combination 4	Bonferoni	Bonferoni	1

Combination 1 resulted in a high false positive rate (24992/25000). For Combination 2,

3, 4, the false positive rate is smaller than 0.05, which are acceptable strategies in controlling false positive rates. It is noteworthy that when adjustment is too stringent, high false negatives can be resulted in the real data. Among the three acceptable strategies, we choose a less stringent Combination 2, where Combination 2 and Combination 3 are equivalent in term of number of false positives.

Supplementary Figures

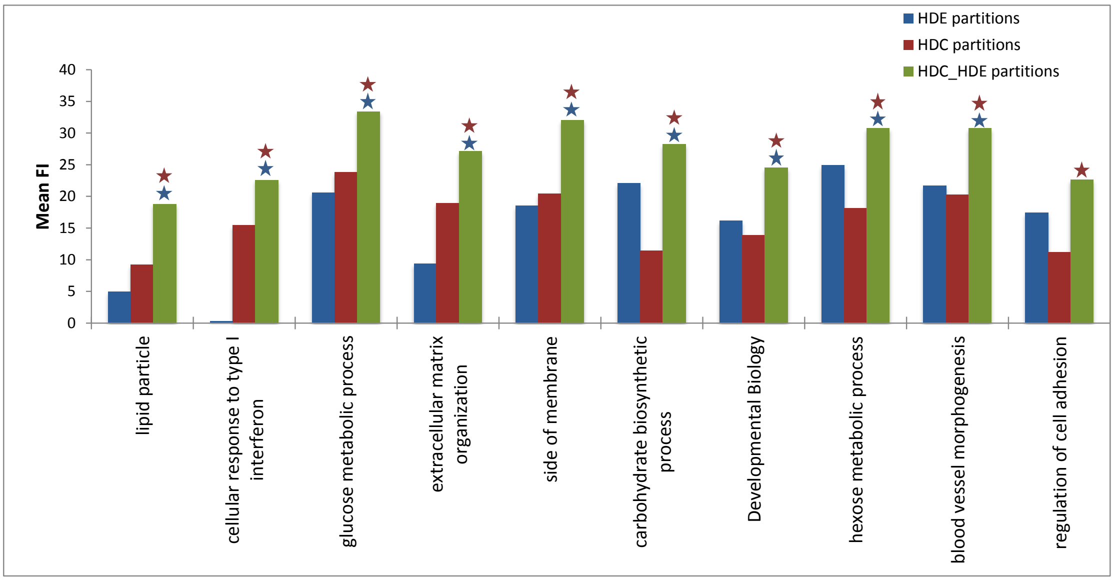

Figure S1. Top 10 best associated gene sets with highest mean minimum functional information (*FI*) gain, $\overline{\Delta_G^*}$, for HDC_HDE partitions in breast cancer data (*validation vs. normal* set).

The HDC_HDE partitions (in green) yield significantly higher mean *FI* than HDC partitions (in red) or HDE partitions (in blue) are marked by red or blue asterisks respectively. The combining HDC_HDE criteria outperformed both of the single criteria in nine gene sets (marked by both red and blue asterisks).

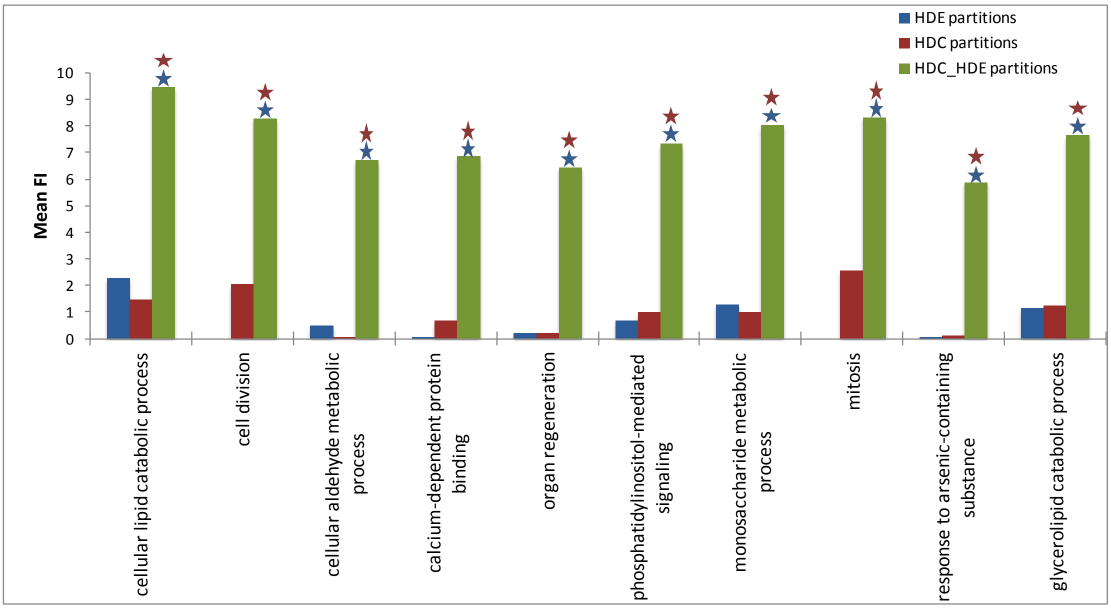

Figure S2. Top 10 best associated gene sets with highest mean minimum functional information (*FI*) gain, $\overline{\Delta_G^*}$, for HDC_HDE partitions in Malaysian breast cancer data.

The HDC_HDE partitions (in green) yield significantly higher mean *FI* than HDC partitions (in red) or HDE partitions (in blue) are marked by red or blue asterisks respectively. The combining HDC_HDE criteria outperformed both of the individual criteria in ten gene sets (marked by both red and blue asterisks).

Supplementary Tables

Table S1. Top 30 best associated gene sets with highest mean minimum functional information (*FI*) gain, $\overline{\Delta_G^*}$, for HDC_HDE partitions in breast cancer data (*discovery vs. normal* set).

Best associated functional gene set	Gene set id	# of HDC_HDE partitions	Mean FI of			Adjusted p-value for test of difference between FI of HDC_HDE partitions		Mean minimum FI gain
			HDE partitions	HDC partitions	HDC_HDE partitions	HDE partitions	HDC partitions	
cellular response to type I interferon	GO\GO:0071357	27	5.4	17.0	28.3	2.0E-06	9.9E-06	10.8
cell-cell junction	GO\GO:0005911	19	5.8	10.9	20.4	5.1E-04	3.6E-03	8.6
lipid particle	GO\GO:0005811	110	11.9	8.9	21.4	4.9E-16	1.9E-17	6.4
regulation of cell adhesion	GO\GO:0030155	38	18.1	11.2	25.2	1.9E-05	9.7E-10	6.0
monocarboxylic acid metabolic process	GO\GO:0032787	15	6.9	14.4	21.4	8.1E-03	2.7E-01	5.7
monosaccharide metabolic process	GO\GO:0005996	16	15.5	11.6	24.6	2.9E-01	4.1E-03	5.7
glucose metabolic process	GO\GO:0006006	49	17.7	16.7	26.2	2.1E-07	7.0E-09	4.9
Developmental Biology	REACTOME\REACT_111045.1	26	18.0	14.4	25.0	3.6E-03	4.0E-06	4.7
regulation of T cell activation	GO\GO:0050863	17	17.8	27.7	33.8	1.4E-02	5.1E-01	3.9
adherens junction	GO\GO:0005912	21	14.6	13.2	21.3	8.9E-03	6.8E-02	3.3
Respiratory electron transport	REACTOME\REACT_22393.2	23	9.2	19.7	24.2	9.5E-05	1.0E+00	3.3
Generic Transcription Pathway	REACTOME\REACT_12627.3	20	2.8	7.0	11.3	1.3E-03	6.5E-01	3.2
Respiratory electron transport, ATP synthesis by chemiosmotic coupling, and heat production by uncoupling proteins.	REACTOME\REACT_6305.3	22	11.3	24.7	28.4	6.4E-05	1.0E+00	3.0
Axon guidance	REACTOME\REACT_18266.1	18	17.8	10.6	22.0	1.0E+00	1.0E-03	3.0
cell junction organization	GO\GO:0034330	15	13.3	18.4	23.2	1.5E-01	9.0E-01	1.9
side of membrane	GO\GO:0098552	119	17.3	34.6	38.2	4.0E-18	8.2E-02	1.9
positive regulation of apoptotic process	GO\GO:0043065	16	21.2	8.4	22.6	1.0E+00	4.1E-03	0.9
small molecule biosynthetic process	GO\GO:0044283	28	15.6	8.9	18.7	1.0E+00	1.7E-04	0.5
carbohydrate biosynthetic process	GO\GO:0016051	41	24.6	12.6	25.4	1.0E+00	3.6E-10	0.3
lytic vacuole	GO\GO:0000323	28	21.3	12.0	23.6	1.0E+00	1.1E-03	0.0
actin binding	GO\GO:0003779	98	25.3	17.3	27.5	1.0E+00	2.6E-14	-0.1
anchoring junction	GO\GO:0070161	31	15.9	18.2	22.2	3.8E-03	1.0E+00	-0.1
hexose metabolic process	GO\GO:0019318	130	23.4	18.7	27.0	8.6E-02	1.2E-12	-0.4
regulation of immune effector process	GO\GO:0002697	29	18.0	31.3	32.3	9.4E-06	1.0E+00	-0.8
mitosis	GO\GO:0007067	221	58.9	23.8	61.7	1.0E+00	4.6E-34	-0.9
regulation of small GTPase mediated signal transduction	GO\GO:0051056	113	30.3	21.9	31.6	1.0E+00	1.2E-15	-1.5
cellular response to cytokine stimulus	GO\GO:0071345	16	26.1	18.0	28.1	1.0E+00	2.2E-01	-1.5
extracellular matrix organization	GO\GO:0030198	36	8.5	29.2	28.6	1.2E-08	1.0E+00	-1.7
lymphocyte activation	GO\GO:0046649	48	9.5	37.0	36.9	4.2E-10	1.0E+00	-1.9
Hemostasis	REACTOME\REACT_604.5	73	28.6	13.4	26.3	1.0E+00	2.3E-11	-2.4

Note:

1. Gene sets with number of significant partitions less than 15 were ignored.
2. In total, 99 unique best associated functional gene sets found.
3. The *p*-values obtained based on Wilcoxon signed-rank test for the difference between *FI* of the HDC_HDE partitions and the *FI* of HDE or HDC partitions are adjusted by Bonferroni corrections.

Table S2. Top 30 best associated gene sets with highest mean minimum functional information (*FI*) gain, $\overline{\Delta_G^*}$, for HDC_HDE partitions in breast cancer data (*validation vs. normal* set).

Best associated functional gene set	Gene set id	# of HDC_HDE partitions	Mean FI of			Adjusted p-value for test of difference between FI of HDC_HDE partitions		Mean minimum FI gain
			HDE partitions	HDC partitions	HDC_HDE partitions	HDE partitions	HDC partitions	
lipid particle	GO\GO:0005811	28	4.9	9.2	18.8	9.2E-07	9.2E-07	8.9
cellular response to type I interferon	GO\GO:0071357	16	0.3	15.4	22.6	3.8E-03	1.9E-02	7.2
glucose metabolic process	GO\GO:0006006	322	20.6	23.8	33.4	2.7E-51	1.3E-51	7.1
extracellular matrix organization	GO\GO:0030198	56	9.4	18.9	27.1	9.5E-09	1.1E-05	6.7
side of membrane	GO\GO:0098552	39	18.5	20.4	32.0	3.9E-06	1.9E-06	6.0
carbohydrate biosynthetic process	GO\GO:0016051	118	22.1	11.4	28.3	3.5E-12	5.2E-19	6.0
Developmental Biology	REACTOME\REACT_111045.1	32	16.1	13.9	24.5	8.0E-07	2.5E-06	4.7
hexose metabolic process	GO\GO:0019318	241	24.9	18.1	30.7	1.5E-22	3.9E-39	4.7
blood vessel morphogenesis	GO\GO:0048514	42	21.7	20.2	30.8	7.0E-05	2.1E-08	4.6
regulation of cell adhesion	GO\GO:0030155	25	17.4	11.1	22.6	9.9E-02	7.3E-06	4.5
Respiratory electron transport	REACTOME\REACT_22393.2	27	9.4	20.6	26.1	3.7E-06	1.8E-02	4.5
regulation of cell activation	GO\GO:0050865	206	18.0	39.7	45.2	4.9E-33	2.6E-11	4.3
regulation of lymphocyte activation	GO\GO:0051249	72	18.5	47.7	51.5	2.1E-11	1.0E+00	3.0
lymphocyte activation	GO\GO:0046649	26	16.5	41.8	45.1	7.3E-06	1.0E+00	2.0
cellular response to cytokine stimulus	GO\GO:0071345	38	26.5	14.0	29.4	1.0E+00	6.3E-09	1.9
leukocyte activation	GO\GO:0045321	22	15.9	26.0	32.7	3.2E-03	5.1E-01	1.5
extracellular matrix	GO\GO:0031012	122	4.7	31.4	32.9	1.2E-19	1.0E+00	1.1
actin binding	GO\GO:0003779	75	22.7	19.0	26.3	7.6E-03	1.1E-07	0.5
negative regulation of intracellular signal transduction	GO\GO:1902532	19	18.8	5.2	19.1	1.0E+00	4.7E-04	0.4
protein catabolic process	GO\GO:0030163	21	22.2	12.0	23.6	1.0E+00	1.2E-03	0.4
positive regulation of cell activation	GO\GO:0050867	21	20.1	31.0	32.2	3.5E-04	1.0E+00	0.1
mRNA processing	GO\GO:0006397	41	17.9	18.4	22.6	2.3E-01	2.5E-01	-0.9
vasculature development	GO\GO:0001944	1459	33.1	28.9	35.3	2.6E-27	4.8E-127	-1.0
Axon guidance	REACTOME\REACT_18266.1	19	16.6	7.4	16.5	1.0E+00	9.4E-04	-1.5
organelle fission	GO\GO:0048285	23	46.7	16.9	45.1	1.0E+00	2.9E-05	-1.6
Generic Transcription Pathway	REACTOME\REACT_12627.3	16	0.5	12.6	10.8	3.8E-03	1.0E+00	-1.9
proteinaceous extracellular matrix	GO\GO:0005578	17	3.3	25.8	24.0	1.9E-03	1.0E+00	-2.2
endocytosis	GO\GO:0006897	22	21.3	6.4	19.0	1.0E+00	5.9E-05	-2.3
actin filament-based process	GO\GO:0030029	126	33.2	18.4	30.3	1.0E+00	4.4E-20	-2.9
actin cytoskeleton organization	GO\GO:0030036	35	29.9	11.7	27.0	1.0E+00	7.2E-09	-2.9

Note:

1. Gene sets with number of significant partitions less than 15 were ignored.
2. In total, 88 unique best associated functional gene sets found.
3. The *p*-values obtained based on Wilcoxon signed-rank test for the difference between *FI* of the HDC_HDE partitions and the *FI* of HDE or HDC partitions are adjusted by Bonferroni corrections.

Table S3. Top 30 best associated gene sets with highest mean minimum functional information (*FI*) gain, $\overline{\Delta_G^*}$, for HDC_HDE partitions in Malaysian breast cancer data.

Best associated functional gene set	Gene set id	# of HDC_HDE partitions	Mean FI of			Adjusted p-value for test of difference between FI of HDC_HDE partitions and FI of		Mean minimum FI gain
			HDE partitions	HDC partitions	HDC_HDE partitions	HDE partitions	HDC partitions	
cellular lipid catabolic process	GO\GO:0044242	28	2.3	1.5	9.4	1.4E-09	3.7E-13	6.4
cell division	GO\GO:0051301	22	0.0	2.1	8.3	1.1E-07	2.8E-08	6.2
cellular aldehyde metabolic process	GO\GO:0006081	18	0.5	0.0	6.7	5.5E-08	2.4E-09	6.2
calcium-dependent protein binding	GO\GO:0048306	20	0.0	0.7	6.8	1.1E-11	1.1E-15	6.2
organ regeneration	GO\GO:0031100	35	0.2	0.2	6.4	6.4E-21	6.7E-21	6.2
phosphatidylinositol-mediated	GO\GO:0048015	43	0.7	1.0	7.3	1.4E-21	3.4E-22	6.0
monosaccharide metabolic process	GO\GO:0005996	15	1.3	1.0	8.0	3.5E-04	2.2E-06	6.0
mitosis	GO\GO:0007067	38	0.0	2.5	8.3	8.3E-19	2.8E-15	5.8
response to arsenic-containing	GO\GO:0046685	16	0.0	0.1	5.9	2.1E-08	1.4E-08	5.7
glycerolipid catabolic process	GO\GO:0046503	16	1.1	1.2	7.7	4.4E-08	8.0E-09	5.7
Lipid and lipoprotein metabolism	REACTOME\REACT_602.8	43	5.2	0.6	10.9	9.6E-14	6.8E-23	5.6
midbody	GO\GO:0030496	16	0.0	2.8	8.2	6.3E-08	6.1E-06	5.4
brown fat cell differentiation	GO\GO:0050873	18	2.8	0.3	8.3	5.3E-07	1.2E-09	5.4
regulation of stress fiber assembly	GO\GO:0051492	18	1.8	0.1	7.3	4.3E-04	2.3E-07	5.4
RAGE receptor binding	GO\GO:0050786	56	0.3	1.0	6.4	3.4E-33	2.1E-34	5.3
C21-steroid hormone metabolic	GO\GO:0008207	17	3.3	0.1	8.6	7.8E-07	4.4E-08	5.3
acylglycerol metabolic process	GO\GO:0006639	19	2.7	0.5	8.2	8.5E-05	1.5E-08	5.3
triglyceride catabolic process	GO\GO:0019433	22	4.0	1.0	9.8	1.5E-06	1.3E-10	5.2
cellular response to lithium ion	GO\GO:0071285	16	2.9	0.9	8.3	1.1E-07	7.3E-07	5.1
response to peptide hormone	GO\GO:0043434	27	2.7	0.0	7.9	5.5E-05	5.4E-12	5.1
monocarboxylic acid metabolic	GO\GO:0032787	75	5.7	1.7	10.9	8.9E-17	2.2E-33	5.1
response to glucocorticoid	GO\GO:0051384	18	2.6	0.1	7.5	3.8E-04	9.5E-08	4.9
retinol metabolic process	GO\GO:0042572	18	1.2	0.2	5.9	5.7E-08	1.8E-10	4.6
monocarboxylic acid binding	GO\GO:0033293	32	2.8	1.0	7.6	8.0E-11	3.7E-15	4.6
myeloid cell differentiation	GO\GO:0030099	15	1.8	0.1	6.2	1.1E-05	2.2E-09	4.4
carboxylic ester hydrolase activity	GO\GO:0052689	52	2.6	0.1	6.9	9.6E-12	2.2E-28	4.2
long-chain fatty acid transport	GO\GO:0015909	44	3.6	0.5	7.7	2.6E-11	5.5E-22	4.0
fatty acid metabolic process	GO\GO:0006631	22	5.2	1.2	9.2	9.5E-02	2.2E-09	4.0
Hormone-sensitive lipase (HSL)-mediated triacylglycerol hydrolysis	REACTOME\REACT_494.1	51	4.3	0.7	8.3	2.2E-11	3.7E-25	4.0
response to reactive oxygen species	GO\GO:0000302	25	4.3	0.4	8.1	8.5E-05	3.8E-11	3.8

Note:

1. Gene sets with number of significant partitions less than 15 were ignored.
2. In total, 54 unique best associated functional gene sets found.
3. The *p*-values obtained based on t-test for the difference between *FI* of the HDC_HDE partitions and the *FI* of HDE or HDC partitions are adjusted by Bonferroni corrections.
